# A New Hope for the Patients of Non‐Alcoholic Steatohepatitis: FDA Gives Green Signal for Resmetirom Use

**DOI:** 10.1002/hsr2.70394

**Published:** 2025-01-22

**Authors:** Abdul Haseeb Hasan, Muhammad Ali Abid, Muhammad Hafi Abid, Laiba Suhail, Abubakar Nazir

**Affiliations:** ^1^ Department of Medicine King Edward Medical University Lahore Pakistan; ^2^ Department of Medicine Faisalabad Medical University Faisalabad Pakistan; ^3^ Oli Health Magazine Organization Kigali Rwanda

**Keywords:** cirrhosis, NAFLD, NASH, non‐alcoholic steatohepatitis, resmetirom, rezdiffra

## Abstract

**Background and Aims:**

Non‐Alcoholic Steatohepatitis (NASH), a severe form of Non‐Alcoholic Fatty Liver Disease (NAFLD), is characterized by inflammation and fibrosis in the liver, often progressing to cirrhosis and hepatocellular carcinoma. Despite its rising prevalence and significant disease burden, effective pharmacological treatments have been limited to lifestyle modifications and surgical interventions. Recently, resmetirom, a thyroid hormone receptor‐β agonist, received FDA approval for treating NASH, offering new hope to patients. This review explores the current understanding of NASH and the role of resmetirom as a breakthrough therapeutic option.

**Methods:**

This study is a comprehensive literature review analyzing peer‐reviewed articles, clinical trial data, and public health reports. No original analyses were conducted, and no statistical software was utilized in this review.

**Results:**

Resmetirom demonstrated efficacy in resolving NASH without fibrosis progression and improving fibrosis scores in patients with biopsy‐confirmed NASH. In a randomized Phase 3 trial, significant histological improvements were observed in 25.9% and 29.9% of patients receiving 80 and 100 mg doses, respectively, compared to 9.7% in the placebo group. Similar trends were noted in fibrosis improvement, with 24.2% and 25.9% of patients showing ≥ 1 stage improvement compared to 14.2% in the placebo group. Adverse effects, including nausea and diarrhea, were reported more frequently in the treatment groups, but the rates of serious adverse events were comparable across groups.

**Conclusion:**

The approval of resmetirom marks a significant advancement in the treatment of NASH, addressing the limitations of lifestyle‐based interventions. As the obesity epidemic drives the increasing prevalence of NASH, resmetirom provides a promising therapeutic option, paving the way for improved patient outcomes and future research.

Non‐alcoholic fatty liver disease (NAFLD), which is characterized by the accumulation of fat in the liver, encompasses two main types: non‐alcoholic fatty liver (NAFL) and non‐alcoholic steatohepatitis (NASH) [1]. NASH, defined by the inflammatory process occurring in the liver due to the accumulation of fats, is a more severe form of NAFLD [2]. These fatty metabolites accumulate in the liver which is the metabolic hub of the body. In the liver, these metabolites cause injury to the cells of the liver, called hepatocytes. The damage to the cells not only promotes fibrotic proliferation in the liver but also contributes to genomic instability. As a result, we are left with a fibrotic liver with decreased function and a greater potential to progress to hepatocellular carcinoma [3]. Approximately one‐fifth of all the patients suffering from NASH develop cirrhosis, making NASH one of the top disorders that require liver transplantation as their treatment [4]. In fact, in the United States, NASH comes second in the list of diseases that necessitate liver transplantation, just behind Hepatitis C [5].

While alcohol is not a contributing factor to NAFLD, it is still closely linked with an unhealthy diet and a sedentary lifestyle, the main forerunners of obesity [2]. Obesity, dyslipidemia, metabolic syndrome, and type 2 diabetes are all closely associated with NASH. Liver biopsy continues to be the gold standard for diagnosing NASH, despite the availability of numerous non‐invasive diagnostics and grading systems for NAFLD and NASH [4]. Global studies indicate that around 25.24% of the population suffers from NAFLD, out of which 59.1% progress to biopsy‐confirmed NASH [6]. It is estimated that approximately 118,000 cirrhosis deaths globally were the result of NASH in 2017 [7]. Talking about the United States in particular, the number of Americans with NASH who have moderate to advanced liver scarring is estimated to range between 6 and 8 million, and the numbers are anticipated to rise, paralleling the rise in obesity in the general population [8].

Moreover, an expanding accumulation of evidence suggests that NAFLD is strongly related to cardiovascular disease (CVD), especially in its severe form [9, 10]. The link can be seen to lie in the factors that contribute to the development of NAFLD and CVD, such as chronic low‐grade inflammation, imbalances in adipokines, and oxidative stress due to reactive oxygen species(ROS) [11]. In NAFLD, inflammatory cytokines like tumor necrosis factor‐alpha (TNF‐α), C‐reactive protein (CRP), and interleukin‐6 (IL‐6) are released, creating a state of inflammation [12]. These cytokines interfere with the production of nitric oxide (NO) by endothelial cells, which leads to endothelial dysfunction [13]. NO plays a crucial role in preventing platelet aggregation by promoting vasodilation through its action on vascular smooth muscle cells. Disruption of NO production can result in vasoconstriction of arteries and lead to reduced vascular perfusion in the myocardium [13]. Another key mechanism linking advanced NAFLD to CVD involves disruptions in methionine metabolism, which occurs primarily in the liver, leading to elevated homocysteine levels (hyperhomocysteinemia) [14]. The elevated homocysteine levels are associated with increased oxidative stress and NAFLD progression [15]. While elevated homocysteine has long been considered a risk factor for CVD and atherosclerosis, its exact role in CVD remains debated and requires further research [16]. However, the combined effects of impaired NO synthesis and oxidative stress due to high homocysteine levels may contribute to CVD development in patients with advanced NAFLD [14]. Additionally, adiponectin, an adipokine that is known to reduce insulin resistance and inflammation, is downregulated in NAFLD and is responsible for the progression of NAFLD to NASH and also to the development of CVD in patients of NAFLD [17]. That is the reason why special attention needs to be paid to NAFLD as a risk factor for CVD‐associated mortality as well.

Previously, many drugs have been tried for the treatment of NASH but they have not fared well in the clinical trials [18]. No drug before Resmetirom has been approved for NASH by the FDA as the other drugs have shown results that were inconclusive and adverse reactions that were poorly tolerated by the patients. Dietary modifications and weight reduction remained the cornerstones for the treatment of NASH, although Vitamin E and pioglitazone were also extensively used [19]. These drugs show some improvement in reducing the inflammatory component of NASH, but they have not shown to reduce the fibrosis component of the disease. Other drugs such as PPAR agonists and GLP‐1 agonists had shown even less of a promise in treatment and some had shown serious adverse effects which had curbed their approval for NASH [18].

The FDA has approved the use of Resmetirom for adults with noncirrhotic NASH with moderate stage to advanced stage of liver fibrosis that are defined as stages F2 to F3 fibrosis. Resmetirom reduces the accumulation of fat in the liver by acting as an agonist of the thyroid hormone receptor (THR‐β) [8, 20]. Resmetirom is to be taken in conjunction with exercise and diet. The dosage is based on the body weight of the patient with patients weighing less than 100 kg to take 80 mg and those weighing 100 kg or more to take 100 mg once daily orally, with or without food. There are no specific contraindications for its use although the liver function tests need to be monitored for any elevations or any side effects related to the liver [21]. The effectiveness and safety of Resmetirom were assessed through an analysis of a primary endpoint after 52 weeks in a double‐blind, placebo‐controlled, randomized Phase 3 trial that spanned 54 months [8]. NASH resolution (including a decrease of at least two points in the NAFLD activity score; scores vary from 0 to 8 where a higher value denotes greater severity) with no worsening of scarring and an improvement (reduction) in fibrosis by ≥ 1 without worsening of the NAFLD activity score were the two main endpoints at 12 months (52 weeks) [22]. Adults with F1B, F2, or F3 fibrosis stages with biopsy‐confirmed NASH were included in the trial. Patients were randomized in a 1:1:1 ratio to receive either a placebo or 80 or 100 mg of resmetirom once a day. The primary analysis population consisted of 966 patients overall, of whom 321 were in the placebo group, 322 in the 80 mg resmetirom group, and 323 in the 100 mg resmetirom group. In comparison to 9.7% of patients in the placebo group, NASH resolution with no progression of scarring was attained by 25.9% of patients and 29.9% of patients in the 80 and 100 mg resmetirom groups respectively (*p* < 0.001 for both comparisons with placebo) [22]. In comparison to 14.2% of patients in the placebo group, 24.2% of patients and 25.9% of patients in the 80 mg and 100 mg resmetirom groups achieved improvement in scarring by ≥ 1 stage with no worsening of the NAFLD activity score (*p* < 0.001 for both comparisons with placebo) [22]. Resmetirom was associated with a higher frequency of nausea and diarrhea than a placebo. Serious adverse event rates were comparable in all study groups: 10.9%, 12.7%, and 11.5% for the 80 mg of resmetirom, 100 mg of resmetirom, and placebo groups respectively. Resmetirom at doses of 80 and 100 mg showed ≥ 1 stage improvement in liver scarring and NASH resolution compared to placebo [22].

A meta‐analysis recently conducted in 2024 that included three randomized control trials and included 2231 patients concluded that resmetirom improved liver enzymes and reduced hepatic fat contents when compared to placebo. Moreover, Resmetirom was generally well‐tolerated and showed no adverse effects. Although nausea and diarrhea were more common in patients taking resmetirom as compared to those taking placebo [23].

From the discussion above, it becomes abundantly clear that NASH cases are expected to rise in the USA as the trend of obesity has been on the rise for the past decade [24]. The digitalization of the world, where it has in one place made lives easier and the world easier to access, on the other hand, it has promoted the adoption of a more sedentary lifestyle and dependence on high‐calorie foods in the country. This has led to an increase in obesity‐associated diseases, as they rise to hold a prominent place in the country's health concerns. It would not be wrong to say that these diseases and their rising incidents did catch the health systems off guard and that they needed more powerful and relevant tools to combat these diseases, especially NASH which has proven to be the beast when it comes to the diseases of the liver. Lifestyle modifications have been the primary treatment offered to the patients of NASH but weight loss has always been something easier said than done, and even if achieved successfully, patients always have a hard time sticking to their low weights and sooner or later indulge in their old habits [25]. Therefore, doctors need to keep in mind the plight of the patients who are trying to lose weight and realize that there are many factors ranging from genetics to hormonal, which can hamper a patient's weight loss and lifestyle modification journey.

Bariatric surgery has also been advised for the severe cases of NASH but like any other surgical procedure, it too is fraught with complications including marginal ulcers, dumping syndromes, and more widely distributed complications of micronutrient deficiencies ranging from neuropathies, anemia, bone‐related issues, poor wound healing to even hair‐loss among others [26, 27]. With the primary goal of reducing weight for NASH, these complications might seem like a pitfall but when weighing the pros and cons, a healthy working liver outweighs them all. But still, a need was felt for an oral medication as well that could improve the outcomes for the patients suffering from NASH. Thus, the approval of resmetirom comes as a huge relief for the patients of NASH.

This orally administered medication is another tool in the arsenal of physicians in treating NASH and ensuring better outcomes for the patients. Resmetirom combined with lifestyle modifications might enable much better results to be obtained for the patients of NASH and that opens up new avenues for future research to explore. The compliance of the treatment regimen with oral medication is expected to be better and last longer than simple advice of lifestyle modifications. It also provides a more comfortable timeline to the patients for their weight loss by halting and even reversing the disease in the liver. The next big step would be to include the drug in the treatment guidelines for NASH and publicize them to doctors and patients alike so that they can be made aware of this newly FDA‐approved drug. Keeping in touch with the latest updates in the field of patient care and treatment is the hallmark of every great physician and a great physician equipped with a great drug would prove a formidable defense against the rising trends of NASH. The price of the drug also needs to be monitored and regularized. Considering the large burden of disease in the country, the drug needs to be made readily available so that all the patients can benefit from it.

To conclude, NASH is one of the most common liver diseases in the USA right now and its rise has paralleled the rise in obesity in the country. FDA has recently approved resmetirom to be used in the country for the treatment of NASH as it has shown great promise in the treatment of the disease. This addition to the treatment modalities of NASH is expected to prove very beneficial for the patients and improve patient outcomes.

## Author Contributions

**Abdul Haseeb Hasan:** conceptualization, project administration, writing–original draft, writing–review and editing, formal analysis, investigation, resources. **Muhammad Ali Abid:** writing–original draft, writing–review and editing. **Muhammad Hafi Abid:** writing–original draft. **Laiba Suhail:** writing–original draft, writing–review and editing. **Abubakar Nazir:** supervision, validation, writing–review and editing, conceptualization.

## Ethics Statement

The authors have nothing to report.

## Conflicts of Interest

The authors declare no conflicts of interest.

## Transparency Statement

The lead author Abubakar Nazir affirms that this manuscript is an honest, accurate, and transparent account of the study being reported; that no important aspects of the study have been omitted; and that any discrepancies from the study as planned (and, if relevant, registered) have been explained.

## Data Availability

The authors have nothing to report.

## References

[1] “Nonalcoholic Fatty Liver Disease (NAFLD) & NASH—NIDDK,” National Institute of Diabetes and Digestive and Kidney Diseases, accessed March 19, 2024, https://www.niddk.nih.gov/health-information/liver-disease/nafld-nash.

[2] J. M. Fraile , S. Palliyil , C. J. Barelle , A. J. Porter , and M. Kovaleva , “Non‐Alcoholic Steatohepatitis (NASH)—A Review of a Crowded Clinical Landscape, Driven by a Complex Disease,” Drug Design, Development and Therapy 15 (September 2021): 3997–4009, https://www.ncbi.nlm.nih.gov/pmc/articles/PMC8473845/. 34588764 10.2147/DDDT.S315724 PMC8473845

[3] S. L. Friedman , B. A. Neuschwander‐Tetri , M. Rinella , and A. J. Sanyal , “Mechanisms of NAFLD Development and Therapeutic Strategies,” Nature Medicine 24, no. 7 (July 2018): 908–922, https://www.ncbi.nlm.nih.gov/pmc/articles/PMC6553468/. 10.1038/s41591-018-0104-9 PMC6553468 29967350

[4] A. C. Sheka , O. Adeyi , J. Thompson , B. Hameed , P. A. Crawford , and S. Ikramuddin , “Nonalcoholic Steatohepatitis: A Review,” Journal of the American Medical Association 323, no. 12 (March 2020): 1175–1183. 32207804 10.1001/jama.2020.2298

[5] Z. Younossi , Q. M. Anstee , M. Marietti , et al., “Global Burden of NAFLD and Nash: Trends, Predictions, Risk Factors and Prevention,” Nature Reviews Gastroenterology & Hepatology 15, no. 1 (January 2018): 11–20. 28930295 10.1038/nrgastro.2017.109

[6] Z. M. Younossi , A. B. Koenig , D. Abdelatif , Y. Fazel , L. Henry , and M. Wymer , “Global Epidemiology of Nonalcoholic Fatty Liver Disease‐Meta‐Analytic Assessment of Prevalence, Incidence, and Outcomes,” Hepatology 64, no. 1 (July 2016): 73–84. 26707365 10.1002/hep.28431

[7] E. B. Tapper , C. Fleming , A. Rendon , et al., “The Burden of Nonalcoholic Steatohepatitis: A Systematic Review of Epidemiology Studies,” Gastro Hep Advances 1, no. 6 (January 2022): 1049–1087. 39131247 10.1016/j.gastha.2022.06.016 PMC11307414

[8] “FDA Approves First Treatment for Patients With Liver Scarring Due to Fatty Liver Disease,” Commissioner of the FDA, 2024, https://www.fda.gov/news-events/press-announcements/fda-approves-first-treatment-patients-liver-scarring-due-fatty-liver-disease.

[9] G. Targher , C. D. Byrne , and H. Tilg , “NAFLD and Increased Risk of Cardiovascular Disease: Clinical Associations, Pathophysiological Mechanisms and Pharmacological Implications,” Gut 69, no. 9 (September 2020): 1691–1705. 32321858 10.1136/gutjnl-2020-320622

[10] S. Ballestri , “Risk of Cardiovascular, Cardiac and Arrhythmic Complications in Patients With Non‐Alcoholic Fatty Liver Disease,” World Journal of Gastroenterology 20, no. 7 (February 2014): 1724–1745. 24587651 10.3748/wjg.v20.i7.1724 PMC3930972

[11] A. Ismaiel and D. L. Dumitraşcu , “Cardiovascular Risk in Fatty Liver Disease: The Liver‐Heart Axis—Literature Review,” Frontiers in Medicine 6 (September 2019): 202. 31616668 10.3389/fmed.2019.00202 PMC6763690

[12] R. Galiero , A. Caturano , E. Vetrano , et al., “Pathophysiological Mechanisms and Clinical Evidence of Relationship Between Nonalcoholic Fatty Liver Disease (NAFLD) and Cardiovascular Disease,” Reviews in Cardiovascular Medicine 22, no. 3 (September 2021): 755–768. 34565074 10.31083/j.rcm2203082

[13] C. Zhang , “The Role of Inflammatory Cytokines in Endothelial Dysfunction,” Basic Research in Cardiology 103, no. 5 (September 2008): 398–406. 18600364 10.1007/s00395-008-0733-0 PMC2705866

[14] T. Pacana , S. Cazanave , A. Verdianelli , et al., “Dysregulated Hepatic Methionine Metabolism Drives Homocysteine Elevation in Diet‐Induced Nonalcoholic Fatty Liver Disease,” PLoS One 10, no. 8 (August 2015): e0136822. 26322888 10.1371/journal.pone.0136822 PMC4556375

[15] C. Matté , F. M. Stefanello , V. Mackedanz , et al., “Homocysteine Induces Oxidative Stress, Inflammatory Infiltration, Fibrosis and Reduces Glycogen/Glycoprotein Content in Liver of Rats,” International Journal of Developmental Neuroscience 27, no. 4 (June 2009): 337–344. 19460627 10.1016/j.ijdevneu.2009.03.005

[16] S. G. Chrysant and G. S. Chrysant , “The Current Status of Homocysteine as a Risk Factor for Cardiovascular Disease: A Mini Review,” Expert Review of Cardiovascular Therapy 16, no. 8 (August 2018): 559–565. 29979619 10.1080/14779072.2018.1497974

[17] S. C. Shabalala , P. V. Dludla , L. Mabasa , et al., “The Effect of Adiponectin in the Pathogenesis of Non‐Alcoholic Fatty Liver Disease (NAFLD) and the Potential Role of Polyphenols in the Modulation of Adiponectin Signaling,” Biomedicine & Pharmacotherapy 131 (November 2020): 110785. 33152943 10.1016/j.biopha.2020.110785

[18] M. Sharma , M. Premkumar , A. V. Kulkarni , P. Kumar , D. N. Reddy , and N. P. Rao , “Drugs for Non‐Alcoholic Steatohepatitis (NASH): Quest for the Holy Grail,” Journal of Clinical and Translational Hepatology 9, no. 1 (February 2021): 40–50. 33604254 10.14218/JCTH.2020.00055 PMC7868704

[19] A. J. Sanyal , N. Chalasani , K. V. Kowdley , et al., “Pioglitazone, Vitamin E, or Placebo for Nonalcoholic Steatohepatitis,” New England Journal of Medicine 362, no. 18 (May 2010): 1675–1685. 20427778 10.1056/NEJMoa0907929 PMC2928471

[20] “Madrigal Pharmaceuticals Announces FDA Approval of Rezdiffra^TM^ (Resmetirom) for the Treatment of Patients With Noncirrhotic Nonalcoholic Steatohepatitis (NASH) With Moderate to Advanced Liver Fibrosis,” Madrigal Pharmaceuticals, accessed March 19, 2024, https://ir.madrigalpharma.com/news-releases/news-release-details/madrigal-pharmaceuticals-announces-fda-approval-rezdiffratm.

[21] “REZDIFFRA (Resmetirom) Tablets, for Oral Use Initial U.S. Approval: 2024,” U.S. Food and Drug Administration, 2024, https://www.accessdata.fda.gov/drugsatfda_docs/label/2024/217785s000lbl.pdf.

[22] S. A. Harrison , P. Bedossa , C. D. Guy , et al., “A Phase 3, Randomized, Controlled Trial of Resmetirom in NASH With Liver Fibrosis,” New England Journal of Medicine 390, no. 6 (February 2024): 497–509, 10.1056/NEJMoa2309000. 38324483

[23] D. Dutta , A. B. M. Kamrul‐Hasan , E. Mondal , L. Nagendra , A. Joshi , and S. Bhattacharya , “Role of Resmetirom, a Liver‐Directed, Thyroid Hormone Receptor Beta‐Selective Agonist, in Managing Nonalcoholic Steatohepatitis: A Systematic Review and Meta‐Analysis,” Endocrine Practice 30, no. 7 (July 2024): 631–638. 38697306 10.1016/j.eprac.2024.04.016

[24] Y. Wang , M. A. Beydoun , J. Min , H. Xue , L. A. Kaminsky , and L. J. Cheskin , “Has the Prevalence of Overweight, Obesity and Central Obesity Levelled Off in the United States? Trends, Patterns, Disparities, and Future Projections for the Obesity Epidemic,” International Journal of Epidemiology 49, no. 3 (June 2020): 810–823. 32016289 10.1093/ije/dyz273 PMC7394965

[25] A. B. Evert and M. J. Franz , “Why Weight Loss Maintenance Is Difficult,” Diabetes Spectrum 30, no. 3 (August 2017): 153–156. 28848306 10.2337/ds017-0025 PMC5556591

[26] S. J. Concors , B. L. Ecker , R. Maduka , et al., “Complications and Surveillance After Bariatric Surgery,” Current Treatment Options in Neurology 18, no. 1 (January 2016): 5. 26860932 10.1007/s11940-015-0383-0

[27] M. L. Collazo‐Clavell and M. Shah , “Common and Rare Complications of Bariatric Surgery,” Endocrinology and Metabolism Clinics of North America 49, no. 2 (June 2020): 329–346. 32418594 10.1016/j.ecl.2020.02.003

